# Systemic dysregulation of TDP-43 binding microRNAs in amyotrophic lateral sclerosis

**DOI:** 10.1186/2051-5960-1-42

**Published:** 2013-07-30

**Authors:** Axel Freischmidt, Kathrin Müller, Albert C Ludolph, Jochen H Weishaupt

**Affiliations:** 1 Department of Neurology, Ulm University, Albert-Einstein-Allee 11, Ulm 89081, Germany

## Abstract

**Background:**

A pathological hallmark of most amyotrophic lateral sclerosis (ALS) cases are intracellular aggregates of the protein TDP-43. The pathophysiological relevance of TDP-43 is underlined by familial ALS cases caused by TDP-43 mutations. TDP-43 is involved in processing of both coding RNAs and microRNAs, which are key epigenetic regulators of transcriptome plasticity and suspected to contribute to neurological diseases. We therefore asked whether the TDP-43 binding microRNAs recently identified in cell lines are also dysregulated in ALS patients. We compared their abundance in cerebrospinal fluid (CSF), serum and immortalized lymphoblast cell lines (LCLs) derived from ALS patients and healthy controls.

**Results:**

We found that expression levels of 5 out of 9 TDP-43 binding microRNAs were altered in the CSF and serum of sporadic ALS cases. The differentially regulated serum microRNAs together with a poor correlation between CSF and serum levels indicate a systemic dysregulation of microRNA abundance independent from the CSF compartment, in line with the ubiquitous expression of TDP-43. The most strongly regulated microRNAs could be confirmed in LCLs from genetically defined ALS patients. While dysregulation of miR-143-5p/3p seems to be a common feature of ALS pathology, downregulation of miR-132-5p/3p and miR-574-5p/3p was evident in sporadic, *TARDBP, FUS* and *C9ORF72,* but not *SOD1* mutant patients. This parallels the TDP-43 pathology found in most ALS cases, but usually not in patients with *SOD1* mutation.

**Conclusions:**

We thus report a systemic and genotype-dependent dysregulation of TDP-43 binding microRNAs in human biomaterial that might reflect an easily accessible biological measure of TDP-43 dysfunction. Furthermore we suggest an independent regulation of TDP-43 binding microRNAs in the serum and CSF compartment as well as a generally low transition of microRNAs across the blood-cerebrospinal fluid barrier.

## Background

Amyotrophic lateral sclerosis (ALS) is a fatal neurodegenerative disease characterized primarily by the progressive loss of spinal motor neurons and cortical pyramidal cells. To date, hundreds of mutations in more than 20 genes have been implicated in the pathology of ALS, whereby mutations in genes coding for superoxide dismutase 1 (SOD1), TAR DNA-binding protein 43 (TDP-43) and fused in sarcoma (FUS) as well as a hexanucleotide expansion on chromosome 9 in open reading frame 72 (*C9ORF72*) account for the most cases with a familial background [1-6]. Despite our growing knowledge about genetics, a vast majority of ALS cases (~85-90%) is considered sporadic (SALS), showing no family history and spontaneous mutations in known causative ALS genes in only a low percentage of patients [7,8]. Moreover, while an increasing number of genetic causes for ALS and other motor neuron disorders have recently been identified, epigenetic factors remain largely undefined with exceptions.

Pathological protein aggregation and/or neuronal cytoplasmic inclusions of SOD1, TDP-43 or FUS are a hallmark of nearly all ALS cases [8,9]. While aggregates of SOD1 and FUS are mostly limited to patients with mutations in the corresponding genes, aberrantly distributed and aggregated TDP-43 is additionally evident in patients with mutations in other genes including *C9ORF72* as well as SALS, further pronouncing the central role of TDP-43 in ALS [1,9,10]. TDP-43 is described as a ubiquitously expressed, multifunctional RNA-binding protein implicated in mRNA transcription and alternative splicing. Although shuffling between the nucleus and the cytoplasm, TDP-43 is located predominantly in the nucleus. When mutated or under conditions of stress, TDP-43 translocates to the cytoplasm where it is hyperphosphorylated and forms insoluble, ubiquitin-positive aggregates [10,11]. The nuclear clearance of TDP-43 as well as the aggregate formation is thought to be involved in ALS pathogenesis.

Recently, TDP-43 was identified as part of nuclear Drosha and cytoplasmic Dicer complexes [12,13] and thus also implicated in microRNA (miRNA) biogenesis [14]. Mature 20–24 nucleotide miRNAs are predominantly negative post-transcriptional regulators of gene expression acting primarily by hybridizing with the 3’ untranslated region of its target mRNAs resulting in translational repression or degradation [15]. Previous studies already showed that miRNA dysregulation can be observed in neurodegenerative disease models [16] including ALS [17]. MiRNA biogenesis starts with long primary transcripts (pri-miRNAs) cleaved by nuclear Drosha complex into shorter miRNA-precursors called pre-miRNAs. The pre-miRNAs are then transported into the cytoplasm where they are further processed by the Dicer complex into mature miRNAs [15]. Before regulating transcriptome plasticity the miRNAs become part of the RNA-induced silencing complex (RISC) facilitating interactions between miRNAs and target mRNAs [18]. It was shown that TDP-43 as part of Drosha and Dicer complexes binds to and promotes the cleavage of selected pri- and pre-miRNAs during their biogenesis. Knock-down experiments in cell lines could confirm 10 mature miRNAs as being dysregulated upon TDP-43 depletion [14,19].

In this study we address the question whether TDP-43 binding miRNAs are actually dysregulated in ALS patients and determined circulating miRNAs in samples of cerebrospinal fluid (CSF) and serum from patients with SALS. We compared miRNA levels between the CSF and the serum compartment and evaluated altered miRNAs as a potential indicator of decreased TDP-43 function in these easy accessible body fluids. Furthermore we could confirm dysregulated TDP-43 binding miRNAs in lymphoblast cell lines (LCLs) derived from SALS patients and genetically defined patients carrying mutations in the genes coding for TDP-43, FUS, SOD1 and C9ORF72, identifying gene specific miRNA alterations.

## Methods

### Patient cohorts and ethics statements

Appropriate approval and procedures were used concerning human subjects. With informed written consent and approved by the national medical ethical review boards in accordance with the Declaration of Helsinki (WMA, 1964), blood samples as well as CSF samples were drawn. CSF and serum samples from the same individuals were derived from 24 healthy controls and 22 ALS patients fulfilling the El-Escorial criteria for definite ALS. Patients were considered sporadic cases (SALS) due to a negative family history and no mutations in known ALS genes (Table 1). There was no correlation between miRNA levels and age (R^2^ ≤ 0,129) or gender.

**Table 1:** Human CSF and serum samples

**Group**	***n***	**Gender**	**Age (mean ± S.D.)**
Controls	24	12 male; 12 female	53.2 ± 16.8
SALS	22	12 male; 10 female	54.5 ± 12.4

### Lymphoblastoid cell lines

Epstein-Barr virus (EBV) transformed lymphoblastoid cell lines (LCLs) were generated from healthy controls as well as sporadic and genetically defined ALS patients. For this study we used LCLs from six healthy controls and eight SALS patients, again with a negative family history and no mutations in known ALS genes. Genetically defined ALS patients carried mutations in the genes coding for TDP-43, FUS, SOD1 or an expanded hexanucleotid repeat in *C9ORF72*. In detail we used LCLs of three TDP-43 mutant (N352S), seven FUS mutant (four K510R, two G478L, one R514G) and five SOD1 mutant (three R115G, two E100K) patients as well as LCLs of seven patients carrying the hexanucleotid repeat expansion in *C9ORF72* with repeat lengths between 620 and 1100 bp (620, 800, 830, 950, 980, 1050 and 1100 bp) as determined by Southern blot analysis.

### RNA isolation

RNA isolation was carried out with the miRNeasy Mini Kit (Qiagen) as specified by the manufacturer. RNA from CSF and serum was isolated in each case from 200 μl using Qiagen’s supplementary protocol for serum and plasma including the spike-in of 5 μl (5 nM) of a synthetic miR-39-3p of *Caenorhabditis elegans* (Cel-miR-39-3p) as a standard for adjusting different RNA isolation and reverse transcription efficiencies.

### Reverse transcription and quantitative PCR

For reverse transcription we used the NCode VILO miRNA cDNA Synthesis Kit (life technologies) according to the manufacturer’s instructions. For CSF and serum samples same volumes of equally isolated RNA were applied to the reactions.

Quantitative PCRs (qPCRs) were run on a CFX96 Real-Time System (Bio-Rad) using the EXPRESS SYBR GreenER qPCR Supermix (life technologies), the Universal qPCR reverse primer included in the reverse transcription kit and miRNA-specific forward primers. MiRNA levels in CSF and serum were normalized relative to Cel-miR-39-3p while expression of miRNAs in LCLs was normalized relative to U6 snRNA using 2^-ΔΔCt^-method [20].

### Statistical analysis

All the statistical analysis was carried out using the two-tailed Mann–Whitney *U* test. *P*-values smaller than 0.05 were considered statistically significant. *: *p* < 0.05; **: *p* < 0.01; ***: *p* < 0.001.

## Results

### TDP-43 binding miRNAs are dysregulated in SALS patients

**Figure 1:**
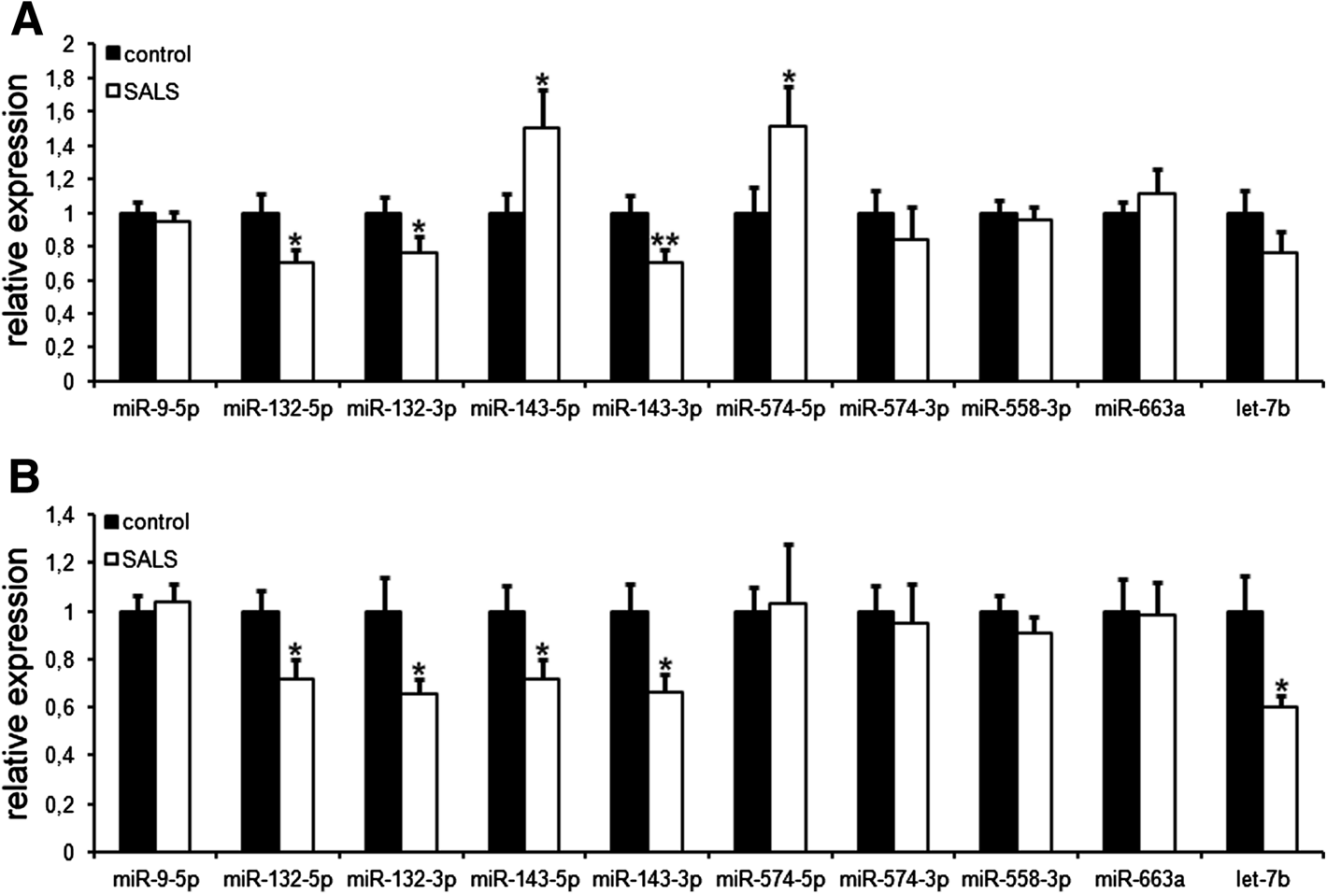
**Dysregulation of TDP-43 binding miRNAs in serum and CSF of SALS patients.** Relative levels of TDP-43 binding miRNAs in CSF **(A)** and serum **(B)** of controls and SALS patients were measured by qPCR. Normalization was performed relative to the spiked-in *C. elegans* miRNA Cel-miR-39-3p, and to the mean expression of the respective miRNA in the control group. Bars indicate mean ± S.E.M.; n = 22 (SALS) or 24 (healthy controls). **(A)** CSF samples of SALS patients exhibit a significant decrease of miR-132-5p, miR-132-3p and miR-143-3p as well as an increase of miR-143-5p and miR-574-5p when compared to healthy controls. The decrease of let-7b did not reach statistical significance but showed a clear trend (*p* = 0.09). **(B)** In serum samples of SALS patients we found a significant decrease of miR-132-5p, miR-132-3p, miR-143-5p, miR-143-3p and let-7b. Only miR-574-3p, miR-558-3p and miR-663a, as well as our control miR-9-5p, showed no significant changes in CSF or serum samples of SALS patients compared to healthy controls.

Recently, 10 miRNAs have been identified that bind to TDP-43 *in vitro* during their biogenesis or in their mature form [14,19]. We measured their relative levels in CSF and serum samples of 22 SALS patients and 24 healthy controls by qPCR. Of the 10 previously identified TDP-43 binding miRNAs miR-181a1-3p was omitted in this study as the effect of a TDP-43 knock-down was only marginal compared to other miRNAs [14]. We additionally studied miR-9-5p, which has multiple functions in neuro-development but does not bind TDP-43 [21]. In CSF samples of SALS patients we found five out of the nine TDP-43 binding miRNAs significantly dysregulated (Figure 1A; Table 2). A significant downregulation was observed for miR-132-5p, -132-3p and −143-3p while miR-143-5p and −574-5p were significantly upregulated.

**Table 2:** Relative miRNA levels in SALS compared to the mean expression in healthy controls

**miRNA**	**Fold change SALS (mean ± S.E.M.)**			
	**CSF**		**Serum**	
miR-9-5p	0.95 ± 0.06	*p* = 0.466	1.04 ± 0.07	*p* = 0.837
miR-132-5p	**0.70** ± **0.07**	***p*** **= 0.047**	**0.72** ± **0.08**	***p*** **= 0.025**
miR-132-3p	**0.76** ± **0.10**	***p*** **= 0.026**	**0.66** ± **0.06**	***p*** **= 0.049**
miR-143-5p	**1.50** ± **0.22**	***p*** **= 0.047**	**0.72** ± **0.08**	***p*** **= 0.048**
miR-143-3p	**0.70** ± **0.08**	***p*** **= 0.006**	**0.66** ± **0.08**	***p*** **= 0.015**
miR-574-5p	**1.51** ± **0.23**	***p*** **= 0.035**	1.03 ± 0.24	*p* = 0.238
miR-574-3p	0.84 ± 0.19	*p* = 0.433	0.95 ± 0.17	*p* = 0.438
miR-558-3p	0.96 ± 0.08	*p* = 0.754	0.91 ± 0.07	*p* = 0.364
miR-663a	1.11 ± 0.14	*p* = 0.826	0.99 ± 0.13	*p* = 0.914
let-7b	0.76 ± 0.13	*p* = 0.090	**0.60** ± **0.05**	***p*** **= 0.033**

Statistical significant differences are highlighted in bold.

In agreement with the systemic expression of TDP-43, we found a similarly strong ALS-associated effect on TDP-43 binding miRNA levels in serum samples of SALS patients, with five out of nine being significantly decreased when compared to healthy controls (Figure 1B; Table 2). Only miR-574-3p, miR-558-3p and miR-663a, as well as our control miR-9-5p that does not bind TDP-43, showed no significant changes in either CSF or serum samples of SALS patients compared to healthy controls. The most robustly regulated miRNAs in our study were miR-132-5p, miR-132-3p and miR-143-3p which were reduced to ~70% relative to healthy controls in both CSF and serum samples. Further miRNAs found to be decreased in the serum were miR-143-5p and let7b.

**Figure 2:**
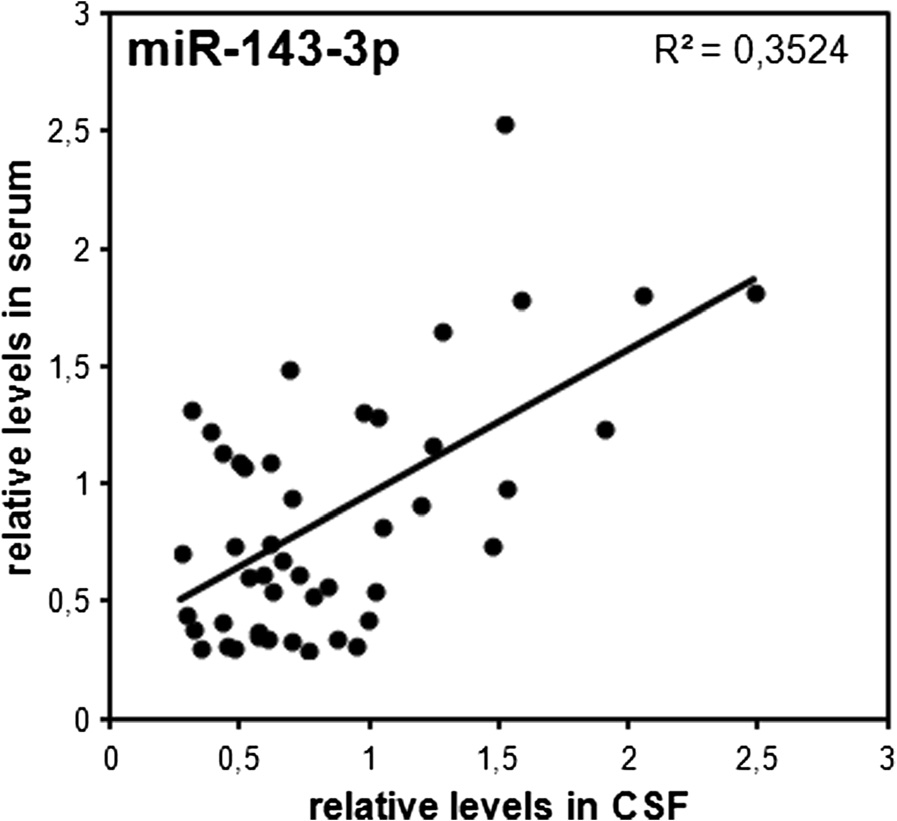
**Correlation of CSF and serum levels of miR-143-3p.** Relative concentrations of miR-143-3p in CSF and serum standardized to the means of the respective compartment are plotted. The plot contains data points from both the healthy controls and ALS patients. A coefficient of determination (*R*^*2*^) of 0.3524 and a *p*-value < 0.01 indicates similar regulation in the CNS and peripheral compartment.

**Figure 3:**
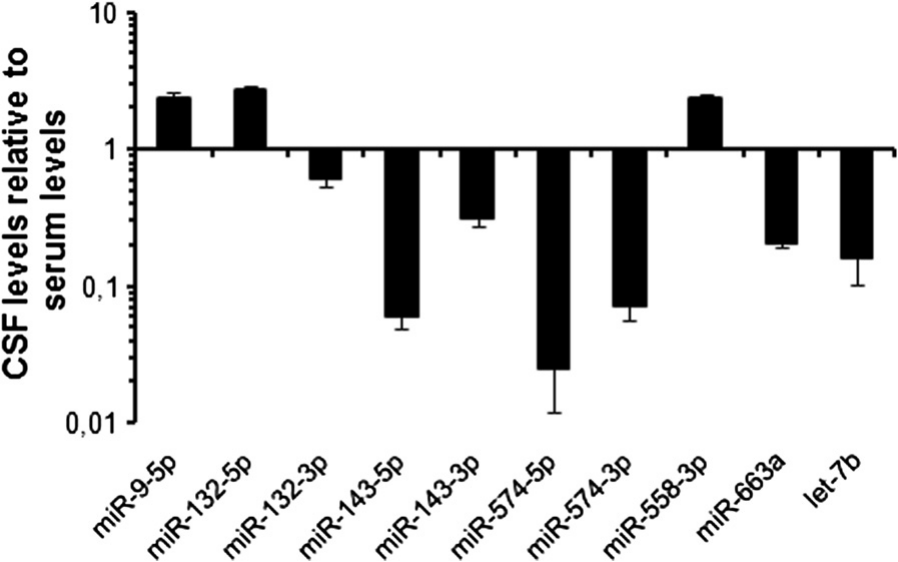
**Logarithmic scale presentation of miRNA levels in CSF compared to serum levels of healthy controls.** While most miRNAs are up to 50-fold less concentrated in CSF samples, miR-9-5p, miR-132-5p and miR-558-3p are 2–3 times more abundant than in serum samples. Bars indicate mean ± S.E.M.

For our study, single CSF and serum samples were derived from the same probands. We were thus able to directly compare CSF and “peripheral” miRNA expression at an individual-to-individual level. Interestingly, only the relative levels of one miRNA (miR-143-3p) were positively correlated between CSF and serum samples (*R*^*2*^ = 0.3524; Figure 2), in contrast to all other miRNAs measured in this study (*R*^*2*^ ≤ 0.166). Moreover, the majority of miRNAs showed an up to approximately 50-fold higher concentration in the serum, while miR-9-5p, miR-132-5p and miR-558-3p were 2–3 times more abundant in the CSF (Figure 3; Table 3). The poor correlation of relative miRNA levels between CSF and serum as well as diverse concentrations and the differential ALS-dependent regulation suggest independent regulatory mechanisms in the CSF and serum compartment.

**Table 3:** Relative miRNA levels in CSF of healthy controls compared to the mean serum levels of the respective miRNA

**miRNA**	**Levels in CSF compared to serum (mean ± S.D.)**	
miR-9-5p	2.32 ± 0.98	*p* = 4.955E-05
miR-132-5p	2.67 ± 0.76	*p* = 1.479E-06
miR-132-3p	0.62 ± 0.31	*p* = 0.14
miR-143-5p	0.06 ± 0.03	*p* = 3.093E-06
miR-143-3p	0.31 ± 0.14	*p* = 4.763E-04
miR-574-5p	0.02 ± 0.04	*p* = 3.093E-06
miR-574-3p	0.07 ± 0.06	*p* = 3.093E-06
miR-558-3p	2.32 ± 0.79	*p* = 2.219E-05
miR-663a	0.21 ± 0.05	*p* = 9.807E-04
let-7b	0.16 ± 0.20	*p* = 8.505E-05

Alterations of miRNA concentrations in CSF and serum were generally not well correlated with ALS-FRS scores of patients, which might be due to our SALS group being almost exclusively at an early stage of the disease (mean ALS-FRS = 39.2 ± 9.2 (mean ± S.D.)).

### Mutation-specific analysis of TDP-43 binding miRNAs in LCLs

**Figure 4:**
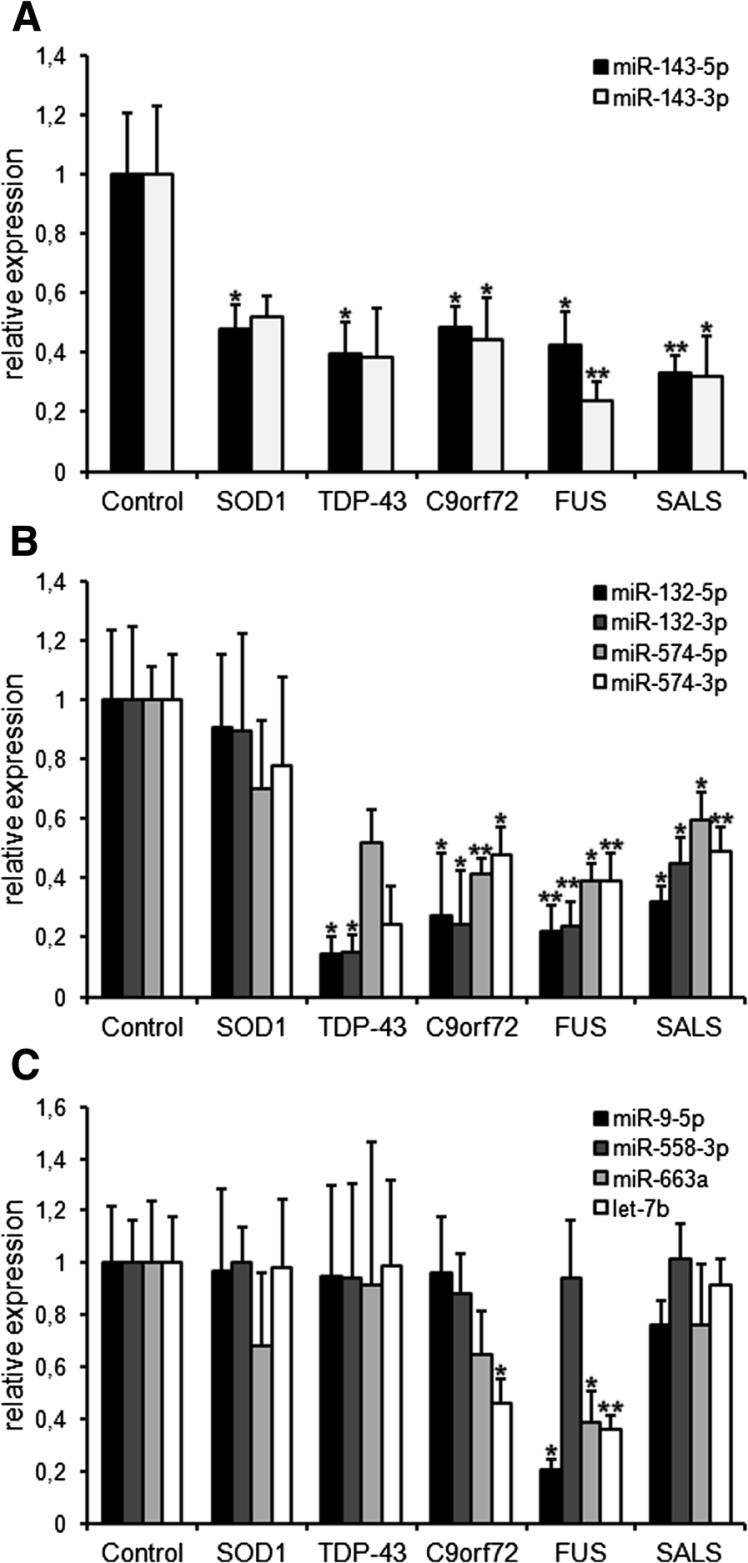
**Levels of TDP-43 binding miRNAs in LCLs of healthy controls and ALS patients.** Genetic backgrounds of the LCLs are indicated by the name of the proteins carrying the mutation, by C9orf72 for LCLs with a hexanucleotide expansion in *C9ORF72* or by SALS for LCLs from sporadic ALS patients with no mutation in known ALS genes. MiRNAs were measured by qPCR and normalized to U6 snRNA. Indicated is the expression of respective miRNAs relative to mean values of the control group. **(A)** Both strands of miR-143 are downregulated in LCLs of all ALS patients tested irrespective of the genetic status. The lack of statistical significance of the decrease of miR-143-3p in *SOD1* and *TARDBP* mutant LCLs is rather due to the small sample number available than to indistinct results. Though, both show a strong trend (*p* ≤ 0.11). **(B)** Both strands of miR-574 and, even more pronounced, of miR-132 are reduced in *TARDBP*, *FUS* and *C9ORF72* mutant LCLs as well as in LCLs derived from SALS patients. In contrary, none of these four miRNAs is altered in *SOD1* mutant LCLs paralleling TDP-43 and FUS pathology. Also here, lack of statistical significance for both strands of miR-574 in *TARDBP* mutant LCLs is due to a small sample number available and results showed a strong trend (*p* ≤ 0.09). **(C)** While miR-558-3p is unaffected throughout all ALS groups, miR-663a and miR-9-5p are exclusively downregulated in *FUS* mutant LCLs. Let-7b levels are significantly reduced in both *FUS* and *C9ORF72* mutant LCLs (bars indicate mean ± S.E.M.; n = 3–8).

To further specify TDP-43 binding miRNAs in ALS we took advantage of lymphoblastoid cell lines (LCLs) generated from healthy controls, SALS patients and genetically defined ALS patients carrying mutations in the genes coding for TDP-43, FUS, SOD1 or the hexanucleotide expansion in *C9ORF72*. Although it is known that immortalization of lymphocytes by EBV transformation is accompanied by specific epigenetic changes, e.g. the regulation of miR-155, the vast majority of miRNAs are most likely not contributing to the transformation process [22,23]. This consideration together with the ubiquitous expression of our genes of interest (*TARDBP*, *C9ORF72*, *SOD1* or *FUS*) provided a rationale to assume that alterations directly induced by mutations in these genes might be represented in LCLs. In addition, the comparison of miRNA levels of ALS-derived LCLs to LCLs from healthy controls further reduced the risk that our observations were due to artifacts of the transformation process. We thus determined the relative levels of TDP-43 binding miRNAs by qPCR, and could reveal mutation-specific as well as mutation-independent changes that partially reflected what we had found in serum and CSF samples (Figure 4; Table 4). A distinctive and mutation-independent decrease to ~50-20% of the level of controls was measured in LCLs of all ALS patients for miR-143-5p and miR-143-3p (Figure 4A), similar to the finding in serum of ALS samples. Also in agreement with the results from ALS patient serum and CSF, both strands of miR-132 showed a similar decrease in LCLs of genetic ALS patients except for those carrying a mutation in the gene coding for SOD1 (Figure 4B). The same pattern of regulation was observed for miR-574-3p and -5p, which were downregulated specifically in LCLs from non-SOD1 cases. Hence, while a decrease of both strands of miR-143 seems to be a common feature of ALS patient derived LCLs, reduced levels of both strands of miR-132 and miR-574 accompany ALS cases with TDP-43 and/or FUS pathology. Dysregulations of further miRNAs measured in this study were gene-specific and almost exclusively restricted to LCLs carrying *FUS*-mutations (Figure 4C). Surprisingly, also the miR-9-5p that was originally chosen as a control miRNA because it does not bind to TDP-43 was drastically reduced in *FUS* mutant LCLs. However, after completion of our experiment and in agreement with this observation, miR-9-5p biogenesis was shown to depend on FUS and miR-9-5p turned out to bind also directly to FUS protein [24].

**Table 4:** Dysregulation of TDP-43 binding miRNAs in lymphoblastoid cell lines (LCLs) derived from sporadic and genetic ALS patients compared to LCLs of healthy controls

**miRNA**	**Sporadic ALS**	**ALS caused by mutation in**			
		**TDP-43**	**SOD1**	**FUS**	***C9ORF72***
miR-132-5p	↓*	↓*	↔	↓**	↓*
miR-132-3p	↓*	↓*	↔	↓**	↓*
miR-143-5p	↓**	↓*	↓*	↓*	↓*
miR-143-3p	↓*	↓^t^	↓^t^	↓**	↓*
miR-574-5p	↓*	↓^t^	↔	↓*	↓**
miR-574-3p	↓**	↓^t^	↔	↓**	↓*
miR-558-3p	↔	↔	↔	↔	↔
miR-663a	↔	↔	↔	↓*	↔
let-7b	↔	↔	↔	↓**	↓*

↔: no change; ↓: downregulation; *,**: level of significance; ^t^: trend.

## Discussion

MiRNAs present in bodyfluids are still controversially discussed concerning origin, function and mode of transport. Nevertheless, circulating miRNAs have been shown to be remarkably stable and to reflect disease conditions designating them as useful biomarkers [25]. Several ALS-related proteins, including TDP-43 that is most central for ALS pathogenesis [8,11,26], have been shown to be involved in miRNA biogenesis [14,19,24]. Specifically in ALS, miRNA alterations are thus most plausible targets for biomarker development or even therapeutic approaches. Several miRNAs depend on TDP-43 for their biogenesis and have been identified as TDP-43 binding in *in vitro* knockdown experiments [14,19]. We now report downregulation of most of these miRNAs in CSF and serum as well as in lymphoblastoid cell lines of sporadic and familial ALS patients.

In conditions of cell stress, in TDP-43 transgenic mouse models or in human ALS post-mortem tissue, wild-type TDP-43 or mutants thereof tend to re-localize from a predominant nuclear localization to the cytoplasm and aggregate in stress granules and/or ubiquitin-positive cytoplasmic inclusions [8,11,27-30]. It seems at least plausible that the reductions in those miRNAs which bind to and require TDP-43 for their biogenesis *in vitro*[14,19] reflect an impaired TDP-43 function or altered subcellular TDP-43 distribution in ALS patients. In line with this consideration is the higher than expected number of regulated miRNAs amongst the TDP-43 binding miRNAs, compared to an unbiased approach: 5 out of 9 miRNAs (56%) were shown to be altered (mostly downregulated) in the CSF and serum, respectively. This is a much higher proportion than found in previous studies looking for regulated miRNAs in e.g. post-mortem brain tissue of patients with frontotemporal lobar degeneration with TDP-43 inclusions [31] or in leucocytes from SALS patients [17] by miRNA arrays. The question whether TDP-43 binding miRNAs are valid surrogate markers for ALS disease progression or useful for diagnosis is beyond the scope of this study and has to be clarified with higher samples numbers and prospective sample collection.

To our knowledge this work is generally the first study comparing CSF and serum miRNAs from samples matched at the level of individual probands. We detected profound “systemic” changes in the serum, which were just as pronounced as miRNA level alterations in the CSF compartment. This surprisingly robust alteration of TDP-43 binding miRNAs outside the brain and CSF compartment is in agreement with the ubiquitous expression and possibly disease-associated “global” functional alteration of TDP-43. Moreover, with the exception of miR-143-3p whose relative abundance showed a clear correlation between the two body fluids both in patients and in controls, the amount of most miRNAs was independently regulated between the two compartments at an individual-to-individual basis. Furthermore, by comparing the miRNA levels in CSF and serum of healthy controls we could show that some miRNAs, like miR-9-5p, miR-132-5p and miR-558-3p are more abundant in the CSF, while others are higher concentrated in serum. Thus, based on the limited number of miRNAs studied in our work, CSF miRNAs do not seem to be a simple mirror of the usually higher abundant serum miRNAs, nor do changes in the serum necessarily reflect alterations of CSF levels, which suggests that transition of miRNAs across the blood-cerebrospinal fluid barrier is not significantly contributing to their abundance in CSF or serum [32].

Nevertheless, systemically dysregulated miRNAs could reflect relevant pathogenic aspects of ALS. Increasing evidence suggests that ALS is a systemic disease, with a “peripheral” pathophysiology, e.g. at the metabolic level [33,34] or with regard to connective tissue [35]. Furthermore, altered serum miRNAs could partially reflect or contribute to peripheral nerve and motor endplate pathology or the failure of regenerative attempts. The latter possibility is suggested by the downregulation of both strands of serum miR-132, which has repeatedly been implicated in neuronal development and synaptogenesis [36]. Moreover, this miRNA (and possibly others) might not only reflect peripheral pathophysiological mechanisms leading to neurodegeneration but could also be a basis for biomarker development in the future as specific molecular markers could turn out to be useful surrogate parameters for disease, even if a correlation between the CSF and serum compartments is not observed.

Overall, our findings point towards a very specific regulation at the level of each miRNA. This is in line with the known high target sequence-dependent, individual role of single miRNAs that can also be tissue or even cell-type dependent. Nevertheless, the fact that we observed mostly a downregulation but rarely an upregulation of TDP-43 binding miRNAs could at least partially be the result of a general default in RNA metabolism in ALS [37].

Transformation of patient-derived lymphocytes by EBV transfection is routinely used to generate immortalized lymphoblastoid cell lines (LCLs) for preservation of DNA. As expected, this transformation process is accompanied and partially depends on epigenetic changes including certain miRNAs, e.g. miR-155 [22,23]. However, lymphocyte transformation seems to depend on specific miRNAs and the majority of miRNAs is most likely not contributing to the transformation process. We thus hypothesized that miRNA alteration resulting from specific mutations in ubiquitously expressed genes (as *TARDBP*, *FUS*, *C9ORF72* or *SOD1*) might still be represented in LCLs. We therefore made an attempt to identify gene specific changes in miRNA levels in LCLs derived from sporadic ALS patients or patients with known gene mutations in *C9ORF72*, *SOD1*, *TARDBP* or *FUS*, which were compared to LCLs derived from healthy control individuals. We found both strands of miR-143 downregulated in all ALS-derived LCLs. As *SOD1*-mutant patients largely lack TDP-43 pathology [9] the underlying mechanism seems to be rather independent of TDP-43 malfunction. Conversely, robust downregulation of both strands of miR-132 and miR-574 are found only in LCLs derived from patients with likely TDP-43 pathology, but not in LCLs carrying a mutation in *SOD1*. We could thus show that the known differences between SOD1 and non-SOD1 ALS regarding TDP-43 pathology [9] are also reflected at the level of TDP-43 binding miRNAs, at least in LCLs.

As TDP-43 and FUS share striking functional and structural similarities [6,8] it is not surprising that mutation of either protein cause similar alterations of miRNA metabolism even in LCLs. Most interestingly, during this study FUS has been implicated in the biogenesis of several miRNAs that overlap with the TDP-43 binding miRNAs found to be downregulated in our study including *FUS* mutant LCLs, e.g. miR-132 and miR-143. Moreover, during our study miR-9-5p biogenesis was shown to depend on FUS [24], providing a plausible explanation why this miRNA, which we had originally chosen as a control miRNA because it does not bind to TDP-43, was drastically reduced in *FUS* mutant LCLs.

Both strands of miR-132, which was the miRNA most robustly downregulated in our study in serum, CSF and LCLs, have been shown to exert multiple functions in neuronal development and morphogenesis. Additionally, downregulation of miR-132 has been detected in brains of patients suffering from Huntington’s and Alzheimer’s disease as well as from schizophrenia and bipolar disorders (summarized in [36]). Importantly, reduced levels of miR-132 have also been found in brains of patients with frontotemporal lobar degeneration with TDP-43 inclusions, a condition closely related to ALS with regard to the molecular pathogenesis and TDP-43 pathology [31]. Thus downregulation of miR-132 seems to be a common feature of several degenerative nervous system conditions. Moreover, even though very speculative, miR-132 downregulation may underline neurodegenerative facets of the psychiatric diseases schizophrenia and bipolar disorder.

Another miRNA found to be dysregulated in all types of samples studied here were both strands of miR-143. This miRNA has not been directly implicated in neurodegeneration so far. However, validated targets of miR-143 comprise proteins involved in cell proliferation and apoptosis [38] as well as multiple proteins participating in actin cytoskeleton remodeling [39]. Recently, experimental and genetic evidence has implicated disturbed actin dynamics in the pathogenesis of motoneuron diseases. For example, mutations in profilin 1, a protein required for actin polymerization, have been shown to cause ALS in a subset of familial cases [40,41].

## Conclusion

In summary, we found that most TDP-43 binding miRNAs were altered both in the CSF and in “peripheral” biomaterial, suggesting a systemic epigenetic dysregulation in ALS. Regulation of a subset of miRNAs paralleled known TDP-43 pathology in respective genetically defined cases. Our findings may be linked to the systemic pathophysiology increasingly reported in ALS and plausibly reflect an altered function of the ubiquitously expressed TDP-43. Consequently, TDP-43 binding serum miRNA levels are candidates for an easily accessible biological measure of TDP-43 dysfunction in ALS. Finally, we provide the first comparison of miRNA expression levels in serum and CSF based on samples matched from the same individuals, which suggests an independent regulation of specific miRNAs in the two compartments and a generally low transition of miRNAs across the blood-cerebrospinal fluid barrier.

## Competing interests

The authors declare that they have no competing interests.

## Authors’ contribution

AF and JHW planned the experiments, interpreted the data and prepared the manuscript. KM genotyped ALS patients. AF carried out the experiments. JHW and ACL supervised the project and gave conceptual input. All authors read and aproved the final manuscript.
